# Predicting the Severity of Parkinson’s Disease Dementia by Assessing the Neuropsychiatric Symptoms with an SVM Regression Model

**DOI:** 10.3390/ijerph18052551

**Published:** 2021-03-04

**Authors:** Haewon Byeon

**Affiliations:** Department of Medical Big Data, College of AI Convergence, Inje University, Gimhae 50834, Gyeonsangnamdo, Korea; bhwpuma@naver.com; Tel.: +82-10-7404-6969

**Keywords:** Parkinson’s disease dementia, instrumental activities of daily living, clinical dementia rating, convergence rate, neuropsychological tests, neuropsychiatric symptoms

## Abstract

In this study, we measured the convergence rate using the mean-squared error (MSE) of the standardized neuropsychological test to determine the severity of Parkinson’s disease dementia (PDD), which is based on support vector machine (SVM) regression (SVR) and present baseline data in order to develop a model to predict the severity of PDD. We analyzed 328 individuals with PDD who were 60 years or older. To identify the SVR with the best prediction power, we compared the classification performance (convergence rate) of eight SVR models (Eps-SVR and Nu-SVR with four kernel functions (a radial basis function (RBF), linear algorithm, polynomial algorithm, and sigmoid)). Among the eight models, the MSE of Nu-SVR-RBF was the lowest (0.078), with the highest convergence rate, whereas the MSE of Eps-SVR-sigmoid was 0.110, with the lowest convergence rate. The results of this study imply that this approach could be useful for measuring the severity of dementia by comprehensively examining axial atypical features, the Korean instrumental activities of daily living (K-IADL), changes in rapid eye movement sleep behavior disorder (RBD), etc. for optimal intervention and caring of the elderly living alone or patients with PDD residing in medically vulnerable areas.

## 1. Introduction

As the survival rate of patients with Parkinson’s disease (PD) has increased and many studies on dementia have been conducted, researchers have become more interested in Parkinson’s disease dementia (PDD). Dementia is a common symptom of patients with PD: As PD progresses, seven out of 10 patients with PD suffer from dementia [1,2]. Moreover, compared to PD without dementia, patients with it have a lower survival rate, a higher risk of experiencing depression [3], and are less responsive to treatment with levodopa (L-DOPA) [4]. Since patients with PDD are more susceptible to the side effects of drugs and their functions deteriorate faster than those with PD without dementia, they require a specialist medical attention [5].

Despite the importance of detecting PDD as soon as possible, it is difficult to accurately screen for it due to three reasons. First, it is difficult to determine whether a decrease in instrumental activities of daily living (IADL), an essential item in the diagnosis of dementia, is caused by a cognitive impairment due to dementia or motor dysfunction due to PD [6]. Second, it is difficult to distinguish whether hallucinations or delusions, the main symptom of dementia, are due to the side effects from the drug being administered or the symptoms of PDD. Third, it is difficult to diagnose PDD in the early stages since patients with PD can have autonomic disturbances, emotional disorders, and/or cognitive impairment [4]. Therefore, selecting a highly sensitive screening test that can accurately discriminate PDD-induced cognitive decline is an important issue that medical professionals are interested in [7].

Meanwhile, evaluating the severity of dementia is critical since only once it has been accurately diagnosed can a physician select the appropriate drugs [8], develop a treatment plan [8], explain the patient’s current condition and offer appropriate caregiving guidelines [9], and discuss prognosis. The clinical dementia rating (CDR) scale [10] has been widely used worldwide as an effective tool for determining the severity of dementia. Although the CDR scale is a commonly-used gold standard, it has several limitations [11]: (1) It takes a lot of time and effort since it must be evaluated through an interview with the guardian; (2) since the questions (items) used to measure the grade (severity of dementia) are inclusive (over a wide range), it is difficult for the medical professional to obtain all of the relevant information about a patient by asking the caregiver to answer these questions; (3) ambiguity can occur since some of the items are too abstract and in some cases, medical professionals cannot judge the progression of dementia; and (4) it does not reflect fine changes in the patient’s condition. Most of all, evaluating the CDR scale results requires a specialist, but elderly people living alone or in medically vulnerable areas often have poor access to medical care [5]. Consequently, if it is possible to predict the CDR scale result for a moderate level of PDD solely using the results of a standardized neuropsychological examination without an interview with the guardian, it will help greatly in identifying the severity of dementia in individuals from medically vulnerable groups such as the elderly living alone.

It has been reported that the severity of dementia is related to demographic factors such as age, the duration of the illness, depression, and motor symptoms such as akinetic-rigidity, and postural instability-gait disturbance, in addition to the neuropsychological profile [12,13]. Therefore, developing a data-mining model that includes these various confounding variables is of great interest and usefulness, and recently, support vector machines (SVMs) have been widely used to explore complex risk factors of diseases [14,15]. The approach has the advantages of less overfitting of probability compared to using decision trees [16] and classifying nonlinear data is possible [17]. Therefore, SVM regression (SVR) was applied to determine the severity of PDD by identifying the convergence rate based on the mean-squared error (MSE) of the standardized neuropsychological test, and baseline data were used to develop a model to predict the severity of PDD.

## 2. Materials and Methods

### 2.1. Data Source

Secondary data were used in the study comprising “Patients with Parkinson’s Disease Dementia Clinical Epidemiology Data (PDE) registry” conducted by the National Biobank of Korea and the Korean Centers for Disease Control and Prevention (K-CDC). The PDE registry comprises nationwide clinical data collected under the supervision of the K-CDC from 14 university hospitals nationwide including those in Seoul and Busan from January to December 2015. The PDE registry includes demographic factors, disease history, health habits, neuropsychological tests, Parkinson’s disease-related motor symptoms, and sleep behavior disorder (SBD) test results (see Byeon et al. [18] for more details). This study was approved by the Research Ethics Review Committee of the National Biobank of Korea and K-CDC (no. KBN-2019-1327; no. KBN-2019-005).

PDD has been designated as idiopathic Parkinson’s disease according to the diagnostic criteria of the United Kingdom Parkinson’s Disease Society Brain Bank [19]. The diagnostic criteria for probable PDD have been suggested by the Bubois et al. [20]. When causes of cognitive impairment other than PD (e.g., hydrocephalus and vascular Parkinsonism) were found in magnetic resonance imaging (MRI) scans, the subject was excluded from the study. Among 335 patients with PDD who were 60 years or older, we excluded seven patients with missing data (non-response or discontinued testing) from the CDR scale data measured by a neurologist and analyzed 328 patients with PDD. Explanatory variables included rapid eye movement (REM), SBDs, PD-related motor signs, demographic variables, disease history, a family history of PD, the Schwab and England Activities of Daily Living (ADL) score [21], the Korean Montreal Cognitive Assessment (K-MoCA) score [22], the Korean Mini-Mental State Examination (K-MMSE) score [23], the Korean IADL (K-IADL) score [24], the Unified Parkinson’s Disease Rating Scale (UPDRS) motor score [25], the UPDRS total score [26], and Hoehn and Yahr (H&Y) stage [27].

### 2.2. Methods

The SVM was operated by finding the most optimal hyperplane that separates data into several classes by applying the maximum margin [28]. For a set of training data where *x_n_* is a multivariate set of *N* observations with observed response values *y_n_* ${\left\{\left(x_{i}y_{i}\right)\right\}}_{i}^{n}$, we apply the regression function f(x) to optimally approximate the given *y* value as follows:

(1) f(x)=〈w,x〉b;w∈X, b∈R,

*w* and *b* in Equation (1) can be optimized via the following transformation:

(2) $$\mathrm{minimize}\;\frac{1}{2}\;{\left\|w\right\|}^{2}+\;C\;\overset{l}{\underset{i=1}{\sum }}\left(\xi_{i}+\xi_{i}^{*}\right)\;\mathrm{subject}\;\mathrm{to}\;\{\begin{array}{c} y_{i}-\;\langle w,x_{i}\rangle \;+b\;\leq \epsilon +\xi_{i}\\ \langle w,\;x_{i}\rangle -b-y_{i}\leq \epsilon +\xi_{i}+\xi_{i}^{*}\\ \xi_{i},\xi_{i}^{*}\;\;\geq \;\;\;0\;\;\;\;\;\;\;\;\;\; \end{array},$$

where *C* is a compromise between the empirical error and the general term ($\frac{1}{2}\;{\left\|w\right\|}^{2}$) and ε is an epsilon tube indicating the tolerance of the error. A general constant is used for empirical error estimation and an increase in *C* indicates an increase in the relative weight of the empirical error within the total error. Moreover, if ε is too small, it induces overfitting of the regression model.

The regression function in Equation (1) can be expressed by using Lagrangian multipliers and optimal constraints as follows:

(3) $$f\left(x,a_{i},a_{i}^{*}\right)=\sum_{i=1}^{l}\left(\alpha_{i}-\alpha_{i}^{*}\right)K\left(x,x_{i}\right)+b,$$

where $K\left(x,\;x_{i}\right)$ is a kernel function. Equation (3) effectively evaluates the nonlinear interrelationship between samples of the training data by expressing them in an internal form [28].

We used the R statistical package (version 4.0.1) for all analyses. To identify the SVR with the best prediction power, we compared the classification performance (convergence rate) of eight SVR models (epsilon-SVR (Eps-SVR) and Nu-SVR with four kernel functions (a radial basis function (RBF), linear algorithm, polynomial algorithm, and sigmoid)). At this time, the convergence rate was determined using the MSE, a loss function based on the mean of the squared error (residual) between the predicted value and the actual value as follows:

(4) $$\frac{1}{n}\sum_{i=1}^{n}{\left(y_{i}-t_{i}\right)}^{2}.$$

This measure allows users to evaluate the similarity between the predicted and actual values to assess the predictive power of the regression model: A smaller value indicates a more accurate model.

## 3. Results

### 3.1. The General Characteristics of the Subjects

**Figure 1:**
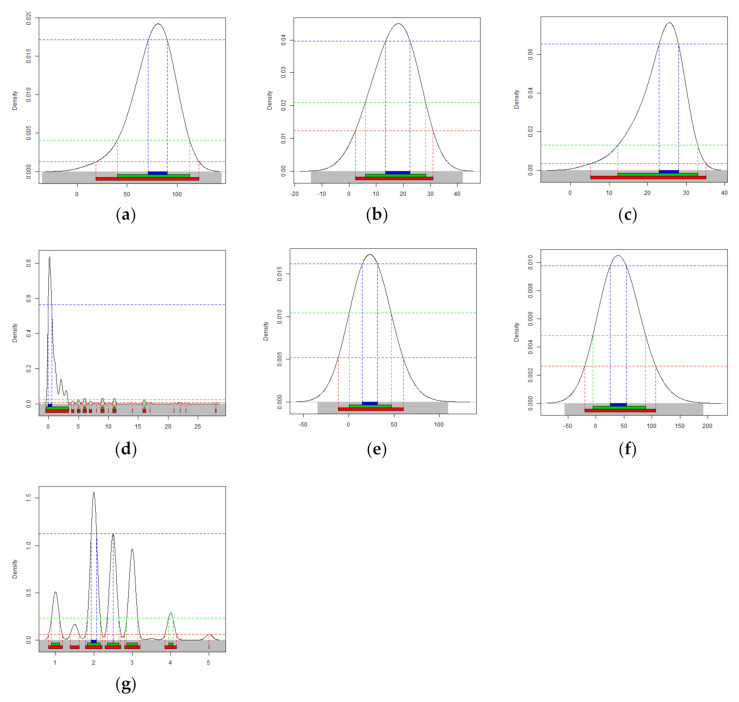
Density plots showing the distribution of the subjects’ neuropsychological test results: (**a**) Schwab and England activities of daily living (ADL) score, (**b**) Korean montreal cognitive assessment (K-MoCA) score, (**c**) Korean mini-mental state examination (K-MMSE) score, (**d**) Korean instrumental activities of daily living (K-IADL) score, (**e**) Unified Parkinson’s disease rating scale (UPDRS) (motor score), (**f**) UPDRS (total score), and (**g**) Hoehn and Yahr (H &Y) stage. The kernel density curve has a probability of 1 if all are added and the curves have been smoothed. The *x*-axis is the score for each test. Dark blue color = 50% highest density interval (HDI); green color = 95% HDI; red color = 99% HDI.

The results of the descriptive analysis on the general characteristics of the 328 PD subjects show that their mean age was 71.9 years old (standard deviation (SD) = 6.1), the mean education period was 7.2 years (SD = 5.0), and the mean age at the time of the initial PD diagnosis was 70.5 years (SD = 6.2). The results also indicate that 75.9%, 17.7%, 4.3%, and 2.1% of the subjects had a CDR of 0.5 or less, 1.0, 2.0, and 3.0 or higher, respectively. Density plots showing the distribution of the subjects’ neuropsychological test results are presented in Figure 1.

### 3.2. Comparing the Convergence Rate of Dementia Severity Prediction Model with the SVR Classification Algorithm

**Figure 2:**
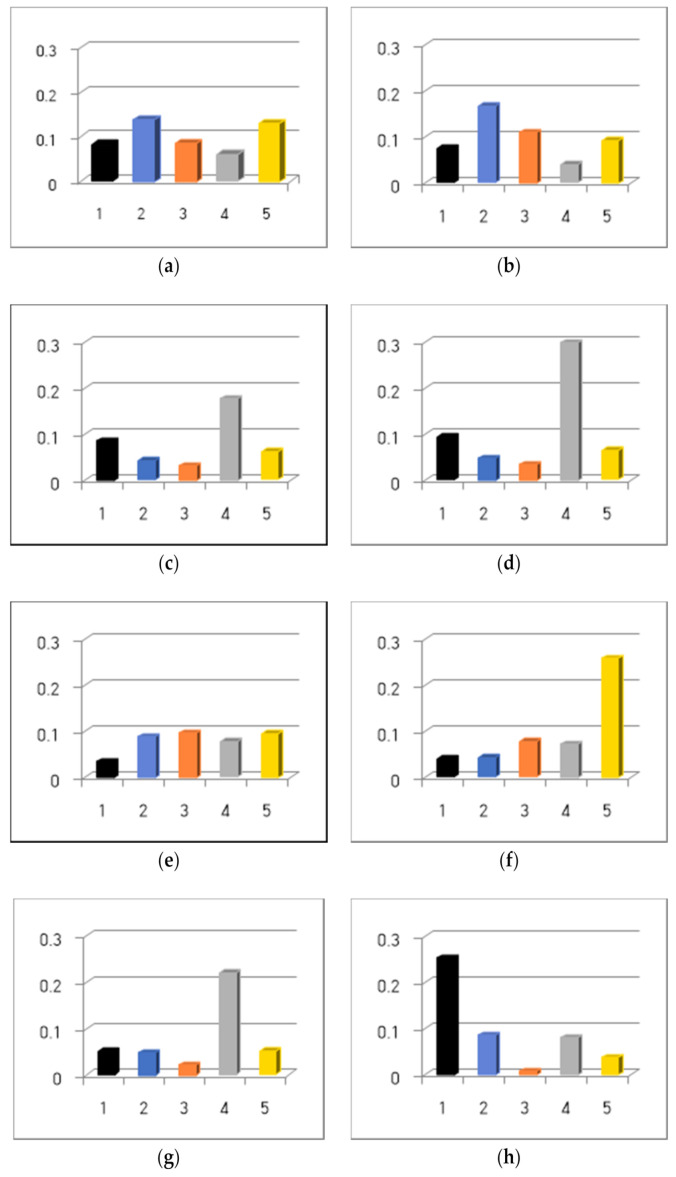
Five-fold cross-validation results of the dementia severity predictive model by the SVR algorithm. (**a**) epsilon-SVR (Eps-SVR)-linear, (**b**) Eps-SVR-polynomial, (**c**) Eps-SVR-radial basis function (RBF), (**d**) Eps-SVR-sigmoid, (**e**) Nu-SVR-linear, (**f**) Nu-SVR-polynomial, (**g**) Nu-SVR-RBF, and (**h**) Nu-SVR-sigmoid.

Since the convergence rate (performance) of the predictive model can be affected by the kernel type, we developed predictive models using Eps-SVM and Nu-SVM with four kernel functions (an RBF, linear algorithm, polynomial algorithm, and sigmoid) to measure the convergence rate according to various kernel types. A comparison of the MSEs of the eight SVMs is reported in Table 1 and Figure 2. The analysis results reveal that the MSE of Nu-SVR-RBF was the lowest (0.078) with the highest convergence rate, whereas the MSE of Eps-sigmoid SVR was 0.110 with the lowest convergence rate.

### 3.3. Factors Related to the Severity of PDD Using the SVR Models

**Figure 3:**
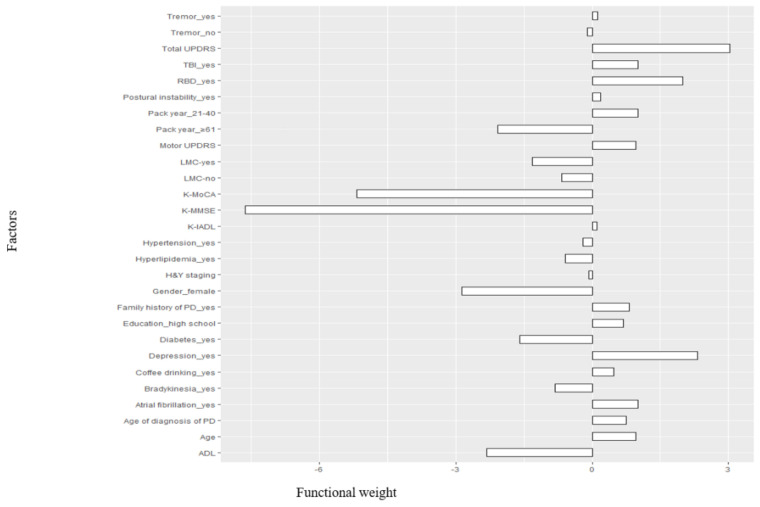
Functional weights of the major variables in the Nu-SVR-RBF model.

We determined that Nu-SVR-RBF with the lowest MSE was the optimal model for predicting the severity of PDD. The functional weight values are presented in Figure 3. Although it is not possible to compare the absolute value of the influence of each factor using the functional weight value, it is possible to determine whether the relationship between the factor and the outcome variable is positive (a risk factor) or negative (a preventive factor). Using 22 support vectors, the Nu-SVR-RBF model showed that K-IADL, total UPDRS, motor UPDRS, tremor, postural instability, age, age at diagnosis of PD, education level (high school graduation or higher), a family history of PD, pack year (21–40), coffee drinker, TBI, atrial fibrillation, RBD, and depression had positive relationships with the severity of dementia.

## 4. Discussion

We developed an SVR-based model for predicting the severity of PDD in patients using data from a nationwide clinical data registry. The results of this study showed that K-IADL, total UPDRS, motor UPDRS, tremor, postural instability, age, age at diagnosis of PD, education level above high school graduation, a family history of PD, pack year (21–40), coffee drinker, TBI, atrial fibrillation, RBD, and depression were major predictors of the severity of PDD. The results of previous studies in which the researchers explored the factors related to PDD reveal that major risk factors and influencing factors inducing PDD can be divided into two groups [29,30]. First, older patients had a higher risk of PDD occurrence and severe cognitive impairment. Second, when the trunk shows axial atypical features including the posture and behavior of the patient as phenotypical of PD, the occurrence of PDD and the severity of cognitive impairment increases [31,32]. Our findings also indicate that PD symptoms such as K-IADL and postural instability, as well as socio-demographic factors such as age, gender, and educational level are indicators of the severity of PDD, which is consistent with the results of [33,34]. Our findings imply that our model could be useful for identifying the severity of dementia by comprehensively examining the axial atypical features, K-IADL, and changes in RBD, etc. for optimal intervention and caring of the elderly living alone or patients with PDD residing in medically vulnerable areas.

PDD, which requires continual treatment, induces a heavy social and economic burden due to caring and medical expenses, and so requires active government support. However, unlike dementia and stroke, the public’s perception of PDD is much lower in South Korea than in other countries such as the US and Japan [35]. To make matters worse, there have only been a few epidemiological studies on PD in South Korea [36] and even fewer on evaluating the relationship between PD symptoms, the cognitive level of PDD, and the severity of PD [37]. Therefore, based on the results of the present study, additional longitudinal studies using a large cohort are required to develop an efficient indicator for predicting the severity of PDD.

Another important finding of this study was that the MSE of Nu-SVR-RBF was the lowest among the convergence rates of eight SVR-based predictive models with four kernel functions (linear, polynomial, RBF, and sigmoid). The performance of SVM is largely dependent on the kernel function and the parameters constituting it [28]. Lamorski et al. [38] also created a Nu-SVM-RBF model with high prediction accuracy. They argued that a linear kernel algorithm with SVM is only suitable when the sample size for the training data items is large and recommended using Nu-regression-RBF when the sample size of the training data is small. Therefore, this was implied that when analyzing data on less than 400 people using SVR (such as the PDD clinical data registry used in this study), developing a predictive model using Nu-SVR-RBF has the highest probability of deriving the best convergence rate.

The importance of this study is that we evaluated the severity of PDD by considering various factors such as the neuropsychological profile, demographic factors, disease symptoms, PD motor problems, and depression. The limitations of the study are as follows. First, although we included general cognitive screening tests such as MMSE and K-MoCA, we did not conduct tests for specific cognitive functions. Since [39] reported a relationship between the deficit of a specific cognitive domain and the progress of PDD, future studies are needed to develop a predictive model for the severity of PDD by including tests for specific cognitive functions such as language and executive functions. Moreover, it is necessary to evaluate the relationship between specific cognitive domains. Second, the sample in this study was not collected by systematic sampling since we used data from hospitals across the country. Hence, we must develop a predictive model by sampling subjects systematically to enable generalization of the results. Third, we did not evaluate biomarkers or genomes. To more sensitively predict the severity of PDD, we must develop a predictive model based on a multi-modal approach that includes genomic data and biomarkers in addition to cognitive tests. Fourth, since this was a cross-sectional study, we could not have identified causal relationships even for factors related to PDD. Further longitudinal studies are needed to prove the causal relationships of the risk and influencing factors identified in this study.

## 5. Conclusions

The CDR scale cannot accurately measure the severity of dementia in the elderly, who have reduced cognitive ability and live alone or in medically vulnerable areas, since it is measured by a specialist based not only by directly interviewing the patient but also collecting the collateral information from the guardian. The results of this study imply that the changes in PD motor symptoms, K-IADL, and RBD could be used as the basis for predicting the severity of PDD. Furthermore, it is necessary to develop a multi-modal screening test that can effectively determine the severity of PDD at an early stage based on the risk and preventive factors derived from the developed predictive model in order to maintain the cognitive health of patients with PD.

## Figures and Tables

**Table 1:** Comparison of the convergence rates of the dementia severity predictive model according to the support vector machine regression (SVR) and kernel function.

SVR	Kernel Function			
	Linear	Polynomial	RBF	Sigmoid
Eps	0.101	0.095	0.079	0.110
Nu	0.079	0.102	0.078	0.091

## Data Availability

Restrictions apply to the availability of these data. Data were obtained from the National Biobank of Korea and are available (from the National Biobank of Korea/http://www.nih.go.kr/NIH/cms/content/eng/14/65714_view.html (accessed on 18 February 2021)) with the permission of the National Biobank of Korea.
